# Association of clinical and laboratory parameters with ambulatory arterial stiffness index in acromegaly patients

**DOI:** 10.12669/pjms.341.14100

**Published:** 2018

**Authors:** Faruk Kilinc, Zafer Pekkolay, Fatih Demircan, Nevzat Gozel, Alpaslan Kemal Tuzcu

**Affiliations:** 1 Faruk Kilinc, Department of Endocrinology, Medical Faculty, Firat University, Elazig, Turkey; 2 Zafer Pekkolay, Department of Endocrinology, Medical Faculty, Dicle University, Diyarbakır, Turkey; 3 Fatih Demircan, Private Etik Life Medical Center, Department of Internal Diseases, Istanbul, Turkey; 4 Nevzat Gozel, Department of Internal Medicine, Medical Faculty, Firat University, Elazig, Turkey; 5 Alpaslan Kemal Tuzcu, Department of Endocrinology, Medical Faculty, Dicle University, Diyarbakır, Turkey

**Keywords:** Acromegaly, Ambulatory arterial stiffness index, Cardiovascular disease, Hypertension

## Abstract

**Objective:**

In this study, we determined the relationship between the ambulatory arterial stiffness index (AASI) and clinical and laboratory parameters in patients with acromegaly.

**Methods:**

Sixty-five patients with acromegaly, who visited to Dicle University Medical Faculty Department of Endocrinology (33 females and 32 males), were included in this study. The study control group consisted of 65 subjects. Demographic and clinical data were recorded. Laboratory data (complete blood count, blood urea nitrogen, creatinine, electrolytes, albumin, lipid profile, growth hormone [GH], insulin-like growth factor-1, and the 75-g oral glucose tolerance test) performed over the last year were evaluated. The AASI was obtained from 24-hour ambulatory blood pressure monitoring records of all patients. This study was completed in 15 months from 2013 to 2015.

**Results:**

Twelve patients (18.4%) had diabetes and 21 patients (32%) had hypertension. The mean AASI value was 0.41 ± 0.14. The mean AASI value in the control group was 0.25 ± 0.09. Growth hormone (GH) levels were positively correlated with the AASI values. AASI values tended to be higher in hypertensive subjects than that in normotensive individuals.

**Conclusions:**

Our results show that the AASI value increased in patients with acromegaly, independent of the increase in blood pressure. The AASI was strongly dependent on the degree of the GH increase in patients with acromegaly and may have an important role predicting cardiovascular risk in patients with acromegaly.

## INTRODUCTION

Acromegaly is a clinical syndrome that develops due to excess release of growth hormone (GH) with an annual incidence of 3–4/1,000,000. The majority of cases (95%) are due to excessive GH secretion from the anterior pituitary.^1^,^2^

Interest in the cardiovascular complications of patients with acromegaly has increased. Cardiac complications include decreased diastolic function with exercise at early and late stages; and cardiomyopathy characterised by cardiac output and advanced congestive heart failure along with dilated cardiomyopathy at later stages.^3^,^4^ Deterioration in cardiac function is an important determinant of mortality in patients with acromegaly.^5^

Over the last decade, cardiovascular research has focused on “arteriosclerosis”, which is a common state of artery hardening in contrast with atherosclerosis, which involves local activity. Influenced by various factors, such as age and high blood pressure, an intense restructuring process takes place in the viscoelastic properties of the large vessel walls, resulting in arteriosclerosis characterised by the formation of hardened arteries, i.e., reduced arterial elasticity or compliance.^6^

As the arteries harden, their width increases and the arterial wall thickens, which is manifested as increased systolic blood pressure (SBP), decreased diastolic blood pressure (DBP), and expanded pulse pressure. These three changes are the main determinants of cardiovascular morbidity and mortality.^6^

Although several measurement methods are currently used to determine arterial stiffness, which is accepted as a strong indication of cardiovascular complications, the challenges and limitations of this method remain controversial.^7^,^8^

A new easy-to-use index has recently been recommended as an indicator of arterial stiffness (decreased arterial elasticity), which can be obtained from arterial blood pressure records and is easy to calculate. The ambulatory arterial stiffness index (AASI) is strongly correlated with conventional measurement methods of arterial stiffness, such as pulse wave velocity (PWV) and the augmentation index (Aix), and provides prognostic information on cardiovascular mortality.^9^,^10^

## METHODS

### Patient Sampling and Exclusion Criteria

All patients diagnosed with acromegaly and under supervision of the Endocrinology and Metabolic Diseases Clinic at Dicle University Medical Faculty Hospital were included in this study. The patients provided written informed consent. This study was approved by the ethics committee of medical research at the university. None of the patients enrolled in the study had advanced cardiovascular or metabolic diseases that could adversely affect the course of the study or the results. The study groups consisted of patients with acromegaly who were hypertensive and normotensive, patients with acromegaly with and without diabetes, patients in and out of remission with acromegaly, and controls. Age, gender, and any previous chronic diseases were recorded. In concordance with the number of patients, 65 subjects were included in the study as controls. The control group was selected among those whose age and gender distribution and metabolic status were close to the enrolled patients without any advanced chronic disease or any history of drug usage that could adversely affect the present study.

### Biochemical and Radiological Measurements

In the endocrinology clinic, the patients were subjected to treatment and follow-up at regular intervals of 1–3 months depending on disease severity. During these check-ups, the patients were requested to provide the relevant examinations, such as GH, insulin-like growth factor-1 (IGF-1), and the 75-g of oral glucose tolerance test (OGTT), a blood glucose-loading test for disease progression and regression, plasma glucose, liver and kidney function tests, and glycated haemoglobin in patients with diabetes.

### Ambulatory Blood Pressure Monitoring

Blood pressure was measured in all patients in an office setting before the start of the ambulatory blood pressure monitoring (ABPM). The ABPM was carried out using a non-invasive blood pressure monitoring device based on an oscillometric method for 24 hour (Oscar 2 24-HR ABP; SunTech Medical, Raleigh, NC, USA). After fitting the device, the patients were allowed to return to their daily routine. The device was programmed to take a blood pressure measurement at 60 minutes intervals. Thus, SBP, DBP, and heart rate were obtained an average of 24 times/day in all patients.

A technically valid reading of at least 80% was required during all registration periods for the operation to be considered successful and this was achieved in all patients. The individual ambulatory AASI values of the patients were calculated from ambulatory blood pressure levels over 24 h using statistical methods. During the index calculations, SBP and DBP from the patients were entered on an individual basis and measured separately.

### Ambulatory Arterial Stiffness Index

The slope of the regression curve obtained from marking the SBP and DBP values from 24- hour blood pressure records of each patient on a (x, y) plane was calculated. The AASI was calculated by the formula: 1 − regression slope. As the stiffness of the arterial system increased, the slope of regression gets closer to zero and the AASI gets closer to 1.

### Statistical Analyses

The statistical evaluation was performed using SPSS 16.0 software (SPSS Inc., Chicago, IL, USA). A p-value < 0.05 was considered significant. The results are expressed as the mean ± standard deviation and percentage (%). Student's *t*-test and the Mann–Whitney *U*-test were used to compare variables with and without a normal distribution, respectively. The relationship between continuous variables was tested by Pearson's correlation analysis, while Spearman's correlation analysis was used to test the relationship between non-parametric variables. This study was completed in 15 months from 2013 to 2015.

## RESULTS

The study included 65 patients, of which 33 were women (50.8%) and 32 were men (49.2%). The mean age of the patients was 43.10 ± 12.2 years. Twelve patients had (18.4%) diabetes mellitus, and 21 patients (32%) had hypertension. The mean age of the control group was 45.14 ± 16.30 years (31 males [47.7%] and 34 females [52.3%]).

The mean and median AASI values of patients were 0.41 ± 0.17 and 0, respectively. The mean AASI value of the control group was 0.24 ± 0.09. The mean AASI value of the patient group (0.41 ± 0.14) was significantly higher than that of the control group (0.24 ± 0.09) (p < 0.001).

A significant positive correlation was detected between the basal GH level and AASI in patients with acromegaly (r = 0.257, p = 0.039). A significant relationship was observed between the AASI and GH level at 90 and 120 minutes of the OGTT (Table-II).

**Table-I:** Comparison of ambulatory blood pressure measurements and average heart rate between the acromegaly and the control groups of patients.

Parameter	Acromegaly patients	Control subjects	P-value
Mean SBP (mmHg)	133.6 ± 24.3 (54–158)	122.83 ± 16.97 (62–142)	0.004*
Mean DBP (mmHg)	81.4 ± 14.2 (44–112)	80.46 ± 16.03 (58–110)	0.72
Day SBP (mmHg)	135.7 ± 18.8 (55–169)	125.61 ± 17.3 (53–146)	0.002*
Day SBP (mmHg)	83.0 ± 16.4 (48–118)	82.38 ± 19.6 (60–104)	0.85
Night SBP (mmHg)	129.5 ± 23.3 (53–150)	114.46 ± 18.4 (62–134)	0.000*
Night DBP (mmHg)	80.6 ± 18.4 (42–106)	73.30 ± 17.8 (59–93)	0.023
Mean heart rate (bpm)	81.5 ± 12.0 (54–102)	82.69 ± 7.8 (60–93)	0.51

SBP: Systolic blood pressure, DBP: Diastolic blood pressure.

**Table-II:** Comparison of the results of the 75-g OGTT between the AASI values of growth hormone (GH) levels in patients with acromegaly.

AASI	GH 0.dk	GH 30.dk	GH 60.dk	GH 90.dk	GH 120.dk
r-value	0.257	0.193	0.235	0.251*	0.280*
P-value	0.039	0.126	0.062	0.046	0.025

OGTT: oral glucose tolerance test GH: growth hormone, AASI: ambulatory arterial stiffness index.

**Table-III:** Measurement of the AASI values of the patient and the control groups.

	Male acromegaly n = 32	Female acromegaly n = 33	Hypertensive acromegaly n = 21	Non-hypertensive acromegaly n = 44	Control group n = 65
AASI	0.40 ± 0.16	0.41 ± 0.13	0.42 ± 0.16	0.40 ± 0.14	0.24 ± 0.09

AASI: ambulatory arterial stiffness index.

No significant difference was found between the genders in patients with acromegaly in terms of the AASI (0.41 ± 0.13 vs. 0.40 ± 0.16, p = 0.98). No correlation was observed between the AASI and age in patients with acromegaly (r = 0.108, p = 0.39).

The AASI value tended to increase with the increase in IGF-1 level (r = 0.15, p = 0.23). The AASI values were not different between hypertensive patients (0.42 ± 0.16) and normotensive patients (0.40 ± 0.140) with acromegaly (p = 0.016).

Of the 65 patients with acromegaly enrolled in this study, 12 had diabetes mellitus with an AASI value of 0.34 ± 0.11. No significant difference was found with the non-diabetic group.

Of the 65 patients, acromegaly was under control in eight patients; however, 57 patients with acromegaly were actively being treated. The AASI values were 0.34 ± 0.07 and 0.42 ± 0.16 in the non-cured group and the group with active disease, respectively (p = 0.28).

## DISCUSSION

Acromegaly is associated with an increased risk of premature mortality. Cardiovascular diseases are the most important cause of the increased mortality in this patient group. Nearly all patients with cardiac disease at the acromegaly diagnosis die within 15 years.^11^ GH level, hypertension, and heart disease constitute the major indicators adversely affecting life expectancy in patients with acromegaly.^12^ In a recent Spanish study of 1,219 cases of acromegaly, 56 deaths occurred, and the most common cause was cardiovascular death.^13^ In patients with acromegaly, cardiovascular disease is expected to be higher due to hypertension (HT), glucose intolerance, and dyslipidaemia.^14^

The prevalence of hypertension was 30% in two large series. Variability in the results may depend on differences in patient selection or technical and environmental factors affecting the blood pressure values during clinical measurements. In a study that investigated the prevalence of hypertension in 40 patients with acromegaly using 24-hour ABPM, the prevalence of hypertension was 42.5% by clinical measurement and 17.5% by the ABPM technique.^15^,^16^ In our study, 32% of patients with acromegaly had hypertension and 18.46% had diabetes mellitus.

Some studies have evaluated arterial stiffness using the AASI, a new index obtained from 24-hour blood pressure records, and it was deemed to be more practical than other measuring methods, which require specialised equipment, and the results were similar and consistent.^17^,^18^

Regression curves for blood pressure have been produced by marking DBP corresponding to SBP values of each individual. Accordingly, they obtained AASI values by extracting the slope of the regression curve from 1.^18^,^19^ Thus, any increase in SBP leads to an increase in the AASI. In our study, the patients with acromegaly were compared with a control group in terms of SBP and DBP values during daytime and nighttime. No significant difference was found in the DBP values; however, a significant increase in SBP was detected in favour of the patients with acromegaly (Table-I). In this study, the mean AASI value (0.41 ± 0.14) of the patients with acromegaly was significantly higher than that of the control (0.24 ± 0.09) and depended on an increase in SBP (p < 0.001).

The mean AASI value obtained from the 24-hour ABPM in our patient group was 0.41 ± 0.14. This value was similar to that reported [0.41 (0.15–0.68)] from a study conducted in 11,291 subjects, which included patients with hypertension, diabetes, and cardiovascular disease.^20^ Again, 95% of the AASI values in normotensive volunteers originating from the Far East and Europe were < 0.55 and 0.57, respectively.^20^ In our study, 95% of the AASI values were < 0.57.

Aortic and carotid artery stiffness measured by PWV increases by approximately 10–15% per decade with age across this population. It has also been suggested that arterial stiffness in women is 5–10% lower compared with that of men of the same age.^21^ A correlation has been reported between arterial stiffness measured by the AASI and age in normotensive healthy and hypertensive subjects^21^-^23^, but the arterial stiffness value according to the AASI is 15% higher than that of men.^20^,^21^ However, Ayşe et al. found no correlation among AASI, age, or gender, which was attributed to a relatively younger patient group (mean age 41 ± 11.98 years).^24^ Surprisingly, we did not find any correlation between AASI and gender in our study, even though our patient group consisted of relatively younger subjects (43.10 ± 12.2 years).

In a study with 11,291 subjects, both the AASI and pulse pressure were higher in women than men, which could have been due to the shorter average stature of women.^25^ In this study, no significant difference was observed in the AASI between men and women.

Dassia et al. reported an assessment of cardiovascular risk factors and 24-hour ABPM in 96 patients with active acromegaly (46 men, mean age 49 ± 14 years). Based on the ABPM measurements, the patients with acromegaly were categorised as normotensive (64 patients) and hypertensive (32 people). Moreover, they were compared to 69 controls consisting of 35 normotensive and 34 hypertensive subjects. Consequently, the AASI values from the patients with acromegaly were higher than those of the controls (p < 0.001). The AASI values of the patients with acromegaly and hypertension were higher compared with those of normotensive subjects (p = 0.01).

A multiple logistic regression analysis demonstrated a significant correlation between the highest AASI value and serum IGF-1 (p = 0.034) across all acromegaly groups.^25^

In our study, the mean AASI in patients with acromegaly (0.41 ± 0.14) was significantly higher than that in the control group (0.24 ± 0.09) (p < 0.001). The AASI values of patients with acromegaly with (21 patients) and without (44 patients) a history of hypertension were 0.42 ± 0.16 and 0.40 ± 0.14, respectively. However, no significant difference (p = 0.016) was found. The AASI values tended to increase with the increase in IGF-1. GH levels increased significantly along with the AASI values.

A significant correlation was found between the AASI values and GH levels measured 90 and 120 minutes after performing the 75-g OGTT in patients with acromegaly. As a result, the values obtained at 90 and 120 minutes could be more significant for predicting the risk of cardiovascular disease.

Similarly, in our study, an increase in the AASI was observed corresponding to an increase in basal GH levels in patients with acromegaly, suggesting the likelihood of increased cardiovascular morbidity and mortality.

Dolan et al. demonstrated in their prospective study that AASI strongly predicts cardiovascular mortality in both hypertensive and normotensive patients. They indicated that the AASI could vary considerably, similar to 24-hour ABPM, but could provide additional hemodynamic information. They asserted that the AASI was a significant indicator of fatal stroke, particularly in young normotensive subjects. Thus, higher AASI values can also be used to predict cerebrovascular mortality.^19^

In their subsequent study, they suggested that the AASI was highly correlated with aortic PWV as well as with central and peripheral Aix, suggesting a novel method for measuring arterial stiffness. Again, this study demonstrated that AASI value was directly proportional to age and mean arterial pressure, but inversely proportional to height, and that AASI values in both women and hypertensive subjects were significantly higher than those in men and normotensive subjects, respectively, if the other variables were held constant.^9^ In our study, the AASI values in patients with acromegaly and a hypertensive history were higher than those in patients with acromegaly without any history of hypertension. In addition, the mean AASI value was higher in women than men.

## CONCLUSIONS

Our study shows that the AASI value increased in patients with acromegaly, independent of the increase in blood pressure. The AASI was strongly dependent on the degree of increase of GH in patients with acromegaly and may have an important role predicting cardiovascular risk in this patient population.

***Author's Contribution:***

**FK and AKT:** Designed and performed the study.

**FK and ZP:** Did data collection and writing of manuscript.

**FD and NG:** Did statistical analysis and editing of manuscript.
